# Women’s Preference for a Male Acquaintance Enhances Social Reward Processing of Material Goods in the Anterior Cingulate Cortex

**DOI:** 10.1371/journal.pone.0136168

**Published:** 2015-08-24

**Authors:** Jun Nakagawa, Muneyoshi Takahashi, Rieko Okada, Eisuke Matsushima, Tetsuya Matsuda

**Affiliations:** 1 Section of Liaison Psychiatry and Palliative Medicine, Graduate School of Medical and Dental Sciences, Tokyo Medical and Dental University, Yushima, Bunkyo-Ku, Tokyo, Japan; 2 Brain Science Institute, Tamagawa University, Machida, Tokyo, Japan; University of Toyama, JAPAN

## Abstract

Men, like the male of many animal species, use gifts to build satisfactory relationships with a desired woman. From the woman’s perspective, all gifts are not always equally rewarding; the reward value of a gift depends on two factors: (1) the giver and (2) the type of the gift (the gift’s social meaning). In this study, we investigated how these two factors interactively determine the reward value of a gift. Specifically, we examined how the neural processing for understanding a gift’s social meaning is modulated by preferences for the giver. We performed a functional magnetic resonance imaging (fMRI) study in which a female participant was asked to judge a gift from a male she was acquainted with in real life. We examined the interactive effects between (1) the female participant’s attitude toward the male acquaintance (liked vs. uninteresting) and (2) the type of the gift (romantic [e.g., bouquet, earrings, and perfumes] vs. non-romantic [e.g., pencils, memo pad, and moneybox]). We found that preference for an acquaintance selectively modulated activity in the anterior cingulate cortex (ACC) in response to romantic gifts, compared to non-romantic gifts. In contrast, if the woman was indifferent toward an acquaintance, no activity modulation was observed in this area for the same gifts. In addition, the ACC showed functional connectivity with the supplementary motor area/dorsal ACC (SMA/dACC), an area within the dorsal mediofrontal cortex, suggesting that it integrates action monitoring and emotional and cognitive processing in decision-making. These results suggest that attitude toward an opposite sex member has a modulatory role in recognizing the social meaning of material goods—preference for the member is a powerful modulator of social reward processing.

## Introduction

Positive interpersonal relationships are crucial for healthy social life, and among them, courtship relationships are one of the most important ones. Human courtship relationships are putatively an evolved form of mammalian mate choice—there are robust biological mechanisms in the human brain that result in a powerful drive to build positive interactions with a desired partner [1]. People experience a complex of affective, cognitive, and rewarding states in courtship relationships [1,2].

Like many male animals that use gifts during courtship and mating [3], men use gifts to build satisfactory relationships with women [4–7]. Gifts are an example of material goods with rewarding properties. In the human brain, neural signals compute these properties to assess motivational relevance in decision-making [8]. Therefore, all received gifts are not equally rewarding—intuitively, the reward value of a gift depends on (1) the giver and (2) the type of the gift. In courtship relationships, the type of a gift can convey the social meaning of romantic intentions from the giver.

Although literature suggests that the reward system is highly involved in human courtship relationships [2,9–11], little is known about the neural processing for the social meaning of material goods in preference-related decision-making. A person’s preference for others affects many social judgments. For instance, attractive models shown in advertisements influence consumer buying behavior [12,13]. Neuroimaging studies have revealed that the subcortical and prefrontal cortical areas are engaged in preference-related decision-making, such as facial attractiveness [14], trustworthiness [15], and appropriate punishment [16,17].

Regarding the social meaning of gifts, people acknowledge the meaning independent of the giver: a gift *per se* can be considered romantic when it contains more personal or love-expressing contents (e.g., cosmetics, perfume, roses, and chocolates) [6,18–20], and it can be non-romantic when it contains more impersonal or non-love—expressing contents (e.g., snacks, drinks, coupons, and CDs) [6,21]. People use non-romantic gifts to reinforce social solidarity or to conform to a sense of obligation [21,22].

Considering these two factors, we assumed that women’s preferences for the giver affect their understanding of the social meaning of gifts. In this study, we investigated how the two factors (giver and type of gift) interactively determine the neural reward value of the gift. Particularly, we examined how preference for a giver affects the neural processing for understanding a gift’s social meaning. We performed fMRI in a novel setting in which female participants were asked to judge the value of gifts from two male acquaintances they knew in real life: for each female participant, one male giver was preferred, while she was indifferent toward the other giver (i.e., the “liked” and “uninteresting” givers). The social meaning of the gift (i.e., gift type) was either “romantic” or “non-romantic.”

We assumed that neural processing is a two-step process; that is, people make judgments after perceiving others, especially their faces [15,23,24]. Therefore, we made the following two predictions regarding the neural processing for understanding a gift’s social meaning. First, a recipient pays attention to who the giver is, and reward-related areas first assess preferences for the opposite sex member. Second, the recipient then considers the social meaning of the gift. The reward-related areas that process preferences for the giver modulate activity in brain areas related to understanding the social meaning of gifts. To our knowledge, this is the first fMRI study showing how preference for an opposite sex member affects the neural processing for understanding a gift’s social meaning.

## Materials and Methods

### Ethics Statement

The study complied with the principles of the Declaration of Helsinki and was approved by respective ethics committees on human research: the Ethics Committee of the Faculty of Medicine, Tokyo Medical and Dental University (No. 1705); and the Ethics Committee of Tamagawa University (N24-4). All participants in this study gave written informed consent prior to participation.

### Participants

Twenty-seven healthy, right-handed Japanese female students from Tamagawa University participated in the fMRI experiment. Their handedness was tested using the Edinburgh Handedness Inventory [25]. Their mean age was 19.81 ± 1.14 (standard deviation, s.d.) years. They reported no psychiatric or neurological medical history, and they were not taking medications that might influence fMRI results. Seven participants were excluded due to excessive movements > 5 mm (4 participants), sleep (2), and poor understanding of the fMRI task (1). Thus, data from 20 participants (mean age = 19.89 ± 1.10 years) were analyzed.

### Stimuli

Before the fMRI experiment, 31 Japanese female students (pre-raters; mean age: 19.90 ± 1.33 years) judged the attractiveness of 120 pictures of material goods on a 9-point scale (1: the least attractive, 9: the most attractive). The pre-raters were age- and education-matched with the fMRI participants—they were from the same university and in the same age group (two-sample *t*-test; fMRI participants, n = 20; pre-raters, n = 31; t_49_ = 0.02, p = 0.98). The pictures of material goods were clothes, sweets, accessories, etc., freely available on the Internet and displayed at 150 × 150 pixel resolution. We chose 60 material goods judged as moderately attractive as target stimuli (mean attractiveness = 5.09 ± 0.57 on the 9-point scale), to avoid a ceiling or floor effect when the fMRI participants judge them as gifts. We also asked the fMRI participants to judge the attractiveness of the 60 goods before the experiment, but at that time, we gave no instruction about the task (judging the gift attractiveness). We confirmed marginal, but not significant, difference in the attractiveness of the material goods *per se* between the fMRI participants and pre-raters (two-sample *t*-test; fMRI participants, n = 20; pre-raters, n = 31; t_49_ = 1.80, p = 0.08).

We then asked the pre-raters to judge the social meaning of each of the 60 material goods on a 3-point scale, if they had received it as a gift from a male. The question was described as below:

Assume that you had received each of the goods as a birthday gift from a male. A gift symbolizes a personal message from the male giver. Do you find the gift romantic? If yes, choose 3; if no, choose 1; and if you are not sure whether it is romantic or not, choose 2.

Based on the rating, we divided the 60 material goods into two groups: 30 romantic and 30 non-romantic gifts (see S1 and S2 Tables). The mean ratings for romantic and non-romantic gifts were 2.44 ± 0.34 and 1.58 ± 0.28, respectively. There was a significant difference in the ratings between the gift types (Mann-Whitney U-test; pre-raters, n = 31; U = 26.50, Z = −6.39, p < 0.001). We used the gifts validated by the pre-raters for the fMRI experiment. The reasons we did not ask the fMRI participants to judge the gift’s meaning are: 1) if we asked them to judge prior to the fMRI experiment, they might have understood what we were interested in testing (the demand characteristics effect), and 2) if we asked them to judge after the experiment, the fMRI task might have affected their judgments (the carryover effect).

Before the task, we asked the fMRI participants to describe two male acquaintances. For each participant, one male acquaintance was preferred (“liked” male), while she was indifferent toward the other male acquaintance (“uninteresting” male). To make the situation as real as possible, we had a short dialogue for at least 5 minutes with the participants and asked what they thought of the liked male. This dialogue was aimed to make the participants to imagine the liked male vividly during the task. All males were university students. The participants knew the liked male for 6.8 ± 3.6 months. Twelve of them were in the lovelorn state and eight of them were dating for less than 6 months. The participants had met the liked males in high school, in a classroom at the same university, or at a club activity inside or outside the campus. We also asked them to draw portraits of each male. Each portrait was displayed at 100 × 100 pixel resolution during the fMRI task.

### Task

Before the task, we asked the fMRI participants to read a setting in which they receive a gift from the liked and uninteresting males. The setting read as follows:

Imagine you are having a birthday party at home. You have two male friends attending the party, one whom you prefer, and the other whom you are indifferent to. Each of them talks to you privately. Each tells you that he brought a birthday gift especially for you. Please judge the attractiveness of the gift that you receive on a 9-point scale, where 1 is the minimum and 9 is the maximum. You should evaluate only the gift, not the giver. Do not imagine that these judgments would change the real relationship between you and the giver. Please concentrate on the judgments and press the button as quickly as possible.

**Fig 1:**
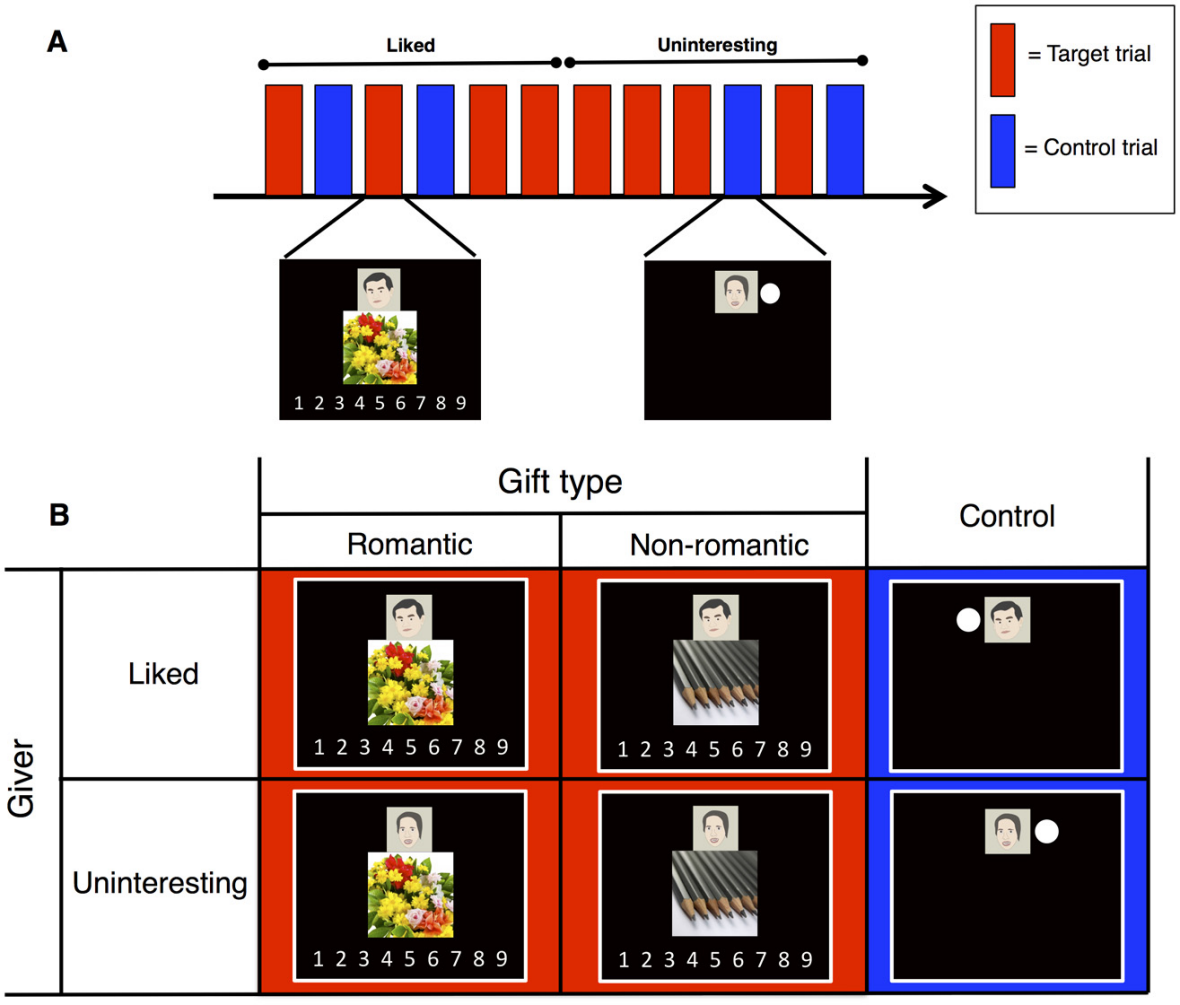
The fMRI task. (A) The task was conducted in an event-related design. Six trials from either the liked or the uninteresting male acquaintance were combined in a set. Each set contained pseudo-randomized four target trials (shown in red) with two control trials (shown in blue). (B) Each trial was created by a 2 × 3 factorial design. In a target trial, female participants judged the gift attractiveness (1: the least attractive, 9: the most attractive). In a control trial, the participants simply pressed the button when a white dot appeared by the side of the portrait. For details, see Materials and Methods.

We used the E-prime 2.0 Professional Software (Psychology Software Tools, Sharpsburg, PA, U.S.A.). We performed the task in an event-related design but we combined six trials from either the liked or the uninteresting giver in a set (Fig 1A)—we wanted to ensure that the participants focus on the gift judgment. Each set contained four target trials (judgments of romantic and non-romantic gifts) mixed with two control trials. The trials were pseudo-randomized, and the order of the sets was counterbalanced across participants. Each trial was followed by the next one after the participants judged the gift's attractiveness. The maximum length of a trial was 6 sec, and a fixation point appeared for 6–10 sec after each trial.

In each target trial, a portrait of a giver, a picture of a gift, and a rating scale were displayed simultaneously (Fig 1B). The participants used a keypad to rate the attractiveness of the gift. The keypad was separated for the left and right hands. The left five fingers corresponded to the ratings 1–5 and the four right fingers, except the little finger, corresponded to the ratings 6–9. The participants had practiced to press buttons correctly before the task. Gift attractiveness and reaction time (RT) for judgments were recorded. We conducted control trials to subtract brain activity on simply viewing the giver and pressing the buttons. In each control trial, a white dot appeared either on the left or on the right side of the portrait (Fig 1B). The participants pressed the button with the left/right index finger if the dot appeared on the left/right.

One task lasted for approximately 30 min. Participants were debriefed at the end of the study. They were also questioned about the task, and they received JP \5,000 (equivalent to US $50).

### Behavioral data analysis

We analyzed behavioral data using the SPSS software (SPSS 19.0 for Mac, SPSS Inc., Chicago, IL, USA). We analyzed the four target conditions created by the 2 × 2 factors (giver × gift type) (Fig 1A). Because participants received the same gifts from each male, we conducted two-way repeated measures analysis of variance (ANOVA) on gift attractiveness and RT. Pairwise comparisons were assessed using the Bonferroni correction [26,27].

### fMRI data acquisition

Functional imaging was conducted using a 3-Tesla Siemens Trio Tim MRI scanner (Erlangen, Germany) at Tamagawa University. For each participant, we acquired whole-brain T1-weighted anatomical scans (repetition time (TR), 2,000 ms; echo time (TE), 1.98 ms; flip angle (FA), 10°; field of view (FOV), 256 mm; matrix, 256 × 256; slice thickness, 1.0 mm; 192 sagittal slices) and gradient echo T2 weighted echo planar images (EPI) with BOLD contrast (TR, 2,500 ms; TE, 25 ms; FA, 90°; FOV, 192 mm; matrix, 64 × 64; slice thickness, 3.0 mm; 42 oblique axial slices). We used a tilted acquisition sequence at 30° to the AC-PC line to prevent signal loss in the medial orbitofrontal cortex [28]. Each brain volume comprised 42 axial slices of 3-mm thickness and 3-mm in-plane resolution. The first five volumes of the images were discarded for equilibration.

### fMRI data preprocessing

Image data were analyzed using SPM8 (Wellcome Department of Imaging Neuroscience, University College London, London, U.K.). Slice-timing correction was initially performed. To correct for participants’ motion, the images were realigned to the mean image. Structural T1 images were co-registered to the mean functional EPI for each participant and normalized to the standard space of the Montreal Neurological Institute (MNI). After normalization, all scans had a resolution of 2 × 2 × 2 mm^3^. Spatial smoothing was applied using a Gaussian kernel with a full-width-at-half maximum (FWHM) of 8 mm.

### Generalized linear model (GLM)

In the first-level analysis, we modeled the four target conditions and two control conditions from each participant, created by the 2 × 3 factorial design (Fig 1B). Six parameters of head motion were entered as regressors of motion correction. High-pass temporal filtering (128 sec) was applied to the data. Because we wanted to remove brain activity that reflected simply viewing the giver and pressing the buttons from the target trials, we made four contrasts by subtracting the control trials. The following four contrasts were calculated for each participant: romantic gifts vs. controls and non-romantic gifts vs. controls for the liked givers, and romantic gifts vs. controls and non-romantic gifts vs. controls for the uninteresting giver. We then conducted second-level random-effects group analysis with the four contrasts using the flexible factorial design. We obtained main effects and pairwise comparisons from the second-level analysis. We also obtained the contrast of the controls (the liked vs. the uninteresting) at the second-level by using one-sample *t*-test. We used a voxel-wise threshold at p < 0.001, uncorrected. We then used a cluster-wise threshold at family-wise error (FWE) p < 0.05, corrected for multiple comparisons to finally identify the significant brain activations. We used Xjview (http://www.alivelearn.net/xjview8) for displaying superimposed significant brain activations with multiple contrasts.

### Region of Interest (ROI) analysis

We used MarsBaR tool for SPM (http://marsbar.sourceforge.net/) to extract activation (parameter estimates) from regions of interest (ROIs). The peak voxel of the ROI was derived from the main effect of the gifts (romantic vs. non-romantic gifts in MNI: –8/40/22). Spherical ROI of 6-mm radius was created for the peak voxel. We analyzed parameter estimates for each of the four conditions. Parameter estimates were extracted from each condition vs. baseline using one-sample *t*-test. We entered parameter estimates of the ROI from each participant into a group-level, to calculate means and standard error means (s.e.m.).

### Psychophysiological Interaction (PPI) analysis

To investigate functional connectivity of the left ACC, we performed a generalized psychophysical interaction (gPPI) analysis. We used a gPPI toolbox (http://www.nitrc.org/projects/gppi) [29] and set the left ACC (peak voxel in MNI: –8/36/30) as a seed region. This peak voxel was derived from the pairwise comparison of the gifts (romantic vs. non-romantic) from the liked giver. Eigenvariate of the peak voxel of the ROI as the voxel of interest at a radius of 6 mm was calculated from each participant. The BOLD signal of the voxel of interest was deconvolved to obtain an estimate of the neuronal signals. Then the neuronal signals from the left ACC seed were multiplied with regressors modeling the task effect and then reconvolved with the canonical HRF. The task effect represents the pairwise comparison of the romantic gift from the liked vs. the uninteresting giver, because we were interested in connectivity in the specific condition in which a participant judges a romantic gift from the liked giver. Model estimation was applied and the resulting SPM showed areas with significant connectivity to the seed. We entered the result of each participant into the second-level random-effects group analysis and used one-sample *t*-test. We used a voxel-wise threshold at p < 0.001, uncorrected; we then used a conservative cluster size criterion with more than 80 contiguous voxels in the cluster. Many PPI studies do not use cluster-wise threshold. Likewise, we used the voxel-wise threshold and cluster size criterion to identify significant functional connectivity.

## Results

### Behavioral results

**Fig 2:**
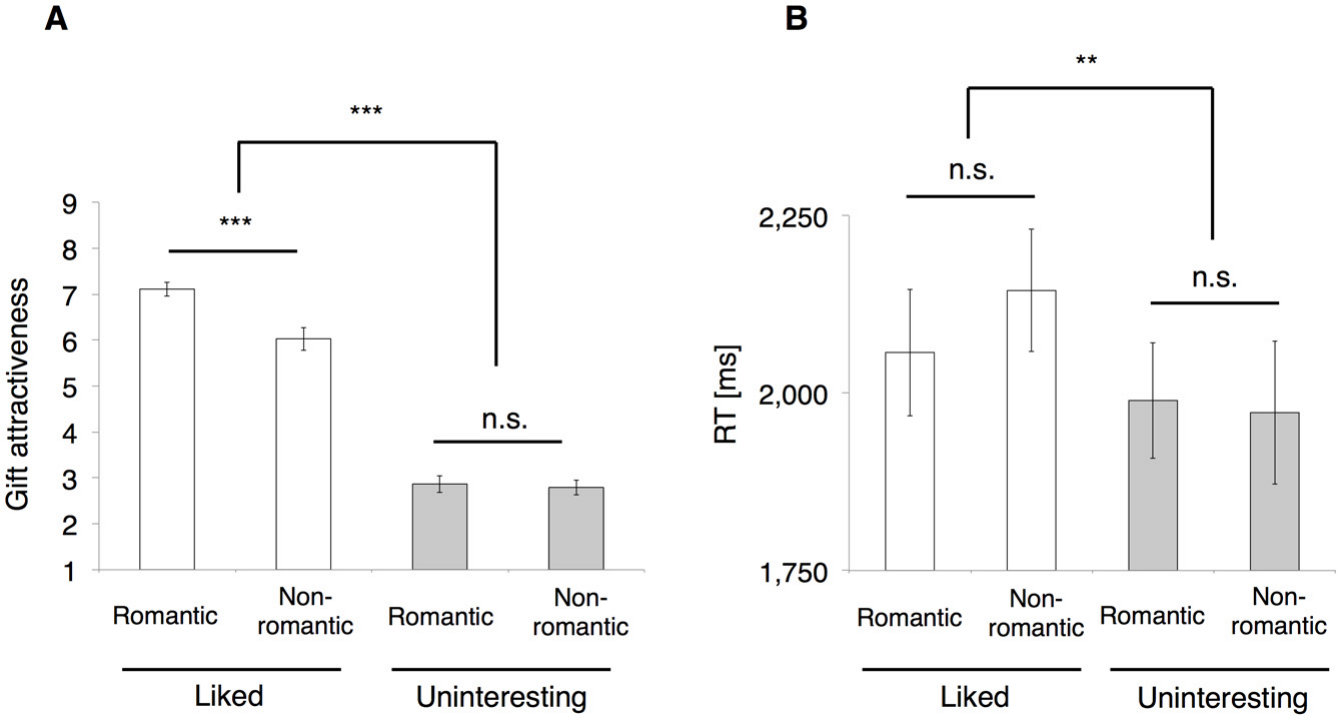
Behavioral results. (A) Mean gift attractiveness across the four target conditions showed significant main effects of both factors and a significant interaction between the two factors (n = 20, two-way repeated measures ANOVA, giver, p < 0.001; gift type, p < 0.01; interaction, p < 0.01). A pairwise comparison showed a difference in attractiveness between gift types for the liked giver (Bonferroni corrected p < 0.001) but not for the uninteresting giver. (B) Mean RT in value judgments across the four target conditions showed only significant main effect of giver (n = 20, two-way repeated measures ANOVA, p < 0.01). Bars show means, and error bars show s.e.m., ** p < 0.01, *** p < 0.001.

The gift attractiveness showed significant main effects of both the giver and gift type (two-way repeated measures ANOVA, giver, F_(1, 38)_ = 458.50, p < 0.001; gift type, F_(1, 38)_ = 7.91, p < 0.01, Fig 2A). The interaction between these two factors was also significant (F_(1, 38)_ = 8.34, p < 0.01). A pairwise comparison of gift attractiveness between the gift types showed a significant difference from the liked giver (Bonferroni corrected p < 0.001, Fig 2A). However, there was no significant difference in the attractiveness from the uninteresting giver (Bonferroni corrected p = 0.76, Fig 2A). This indicates that a romantic gift is more attractive than a non-romantic gift, only when a recipient liked the giver.

The mean reaction time (RT) showed significant main effect of giver but not the gift type (two-way repeated measures ANOVA, giver, F_(1, 38)_ = 8.67, p < 0.01; gift type: F_(1, 38)_ = 0.09, p = 0.77, Fig 2B). The interaction between these factors was not significant (F_(1, 38)_ = 1.64, p = 0.21). A pairwise comparison of the RT between the gift types did not show a significant difference from both givers (the liked, Bonferroni corrected p = 0.49; the uninteresting, Bonferroni corrected p = 0.90, Fig 2B). A recipient spends more time to judge gifts from the liked than the uninteresting giver, regardless of the gift type. We did not counterbalance the keypads for judgments (i.e., the left hand for lower ratings [from 1 to 5]; the right hand for higher ratings [from 6 to 9]). We interpret the short RT from the uninteresting giver that the participants dexterously decided the gift value with their left hands, although they were all right-handed.

### fMRI results

#### Main effects and pairwise comparisons

First, we analyzed the contrast of the control conditions (the liked vs. the uninteresting giver). This is important to validate that the experimental manipulations of the target conditions led to the expected brain activations. There was no significant activation in this contrast: there was no significant difference in brain activation between the liked and uninteresting control conditions.

**Fig 3:**
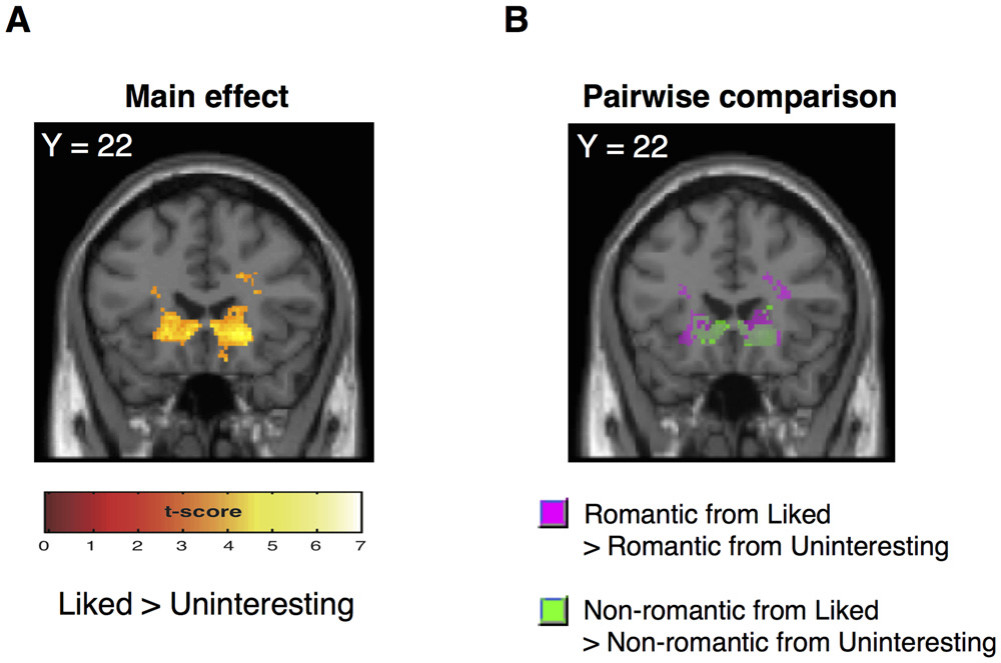
Brain activation for the effect of the givers. (A) Significant activation was found in the bilateral caudate with a main effect of giver (liked vs. uninteresting). (B) Superimposed significant activations in the bilateral caudate for a pairwise comparison between givers for romantic (in purple) and non-romantic (in green) gifts (i.e., romantic/non-romantic gifts from the liked vs. the uninteresting giver). All activations are displayed at a threshold of p < 0.001, uncorrected at the voxel-level.

We found a main effect of giver (liked vs. uninteresting) for activation in the bilateral caudate (Fig 3A, Table 1). We performed a pairwise comparison between the givers with the same gift type (i.e., the romantic/non-romantic gifts from the liked vs. uninteresting giver). We found overlapped activation in the same areas for both pairwise comparisons (Fig 3B). Caudate activation reflects an increase in the gift attractiveness modulated by the liked giver. We also found activations in other brain areas including the left posterior insula, right fusiform gyrus, and right parahippocampus (Table 1).

**Table 1:** Significant brain activations observed in contrast analyses.

Region	Peak in MNI			Z-score	Cluster size
	x	y	z		
*Liked > Uninteresting*					
R Caudate	20	22	−4	4.74	2512
L Caudate	−4	18	−2	4.44	
L Posterior Insula	−34	−16	2	4.95	
L PCG	−38	−28	54	5.90	1685
L MFG	−24	−2	50	3.72	
R Cerebellum	22	−46	−28	5.26	1302
R FG	42	−44	−12	3.81	385
R Parahippocampus	38	−32	−14	3.56	
*Romantic > Non-romantic*					
L ACC*	−8	40	22	3.72	89
*(Romantic > Non-romantic) from Liked*					
L ACC	−8	36	30	3.83	377

R = right, L = left, PCG = postcentral gyrus, MFG = middle frontal gyrus, FG = fusiform gyrus, ACC = anterior cingulate cortex.

*It was significant at the voxel-level but not at the cluster-level.

**Fig 4:**
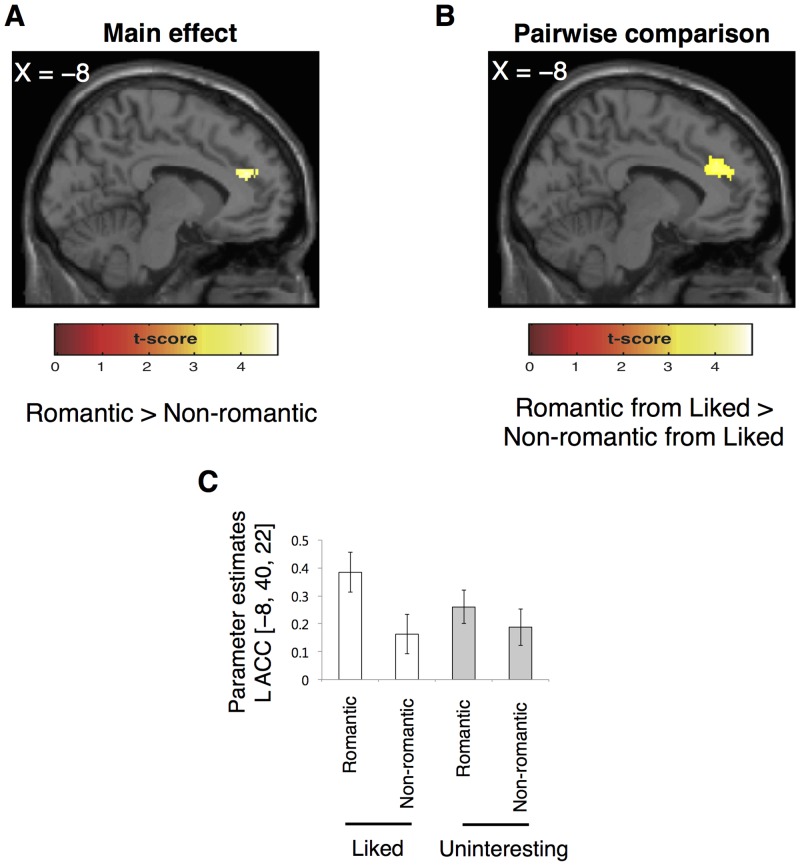
Brain activation for the effect of the gift types. (A) The left ACC showed a significant main effect of the gift type (romantic vs. non-romantic) at the voxel-level but not at the cluster-level. (B) The left ACC showed significant difference both at the voxel- and cluster-level for a pairwise comparison between the gift types from the liked giver. (C) Parameter estimates of the ROI in the left ACC across the four target conditions. The peak voxel is derived from the main effect of the gift types. All activations are displayed at a threshold of p < 0.001, uncorrected at the voxel-level. Parameter estimates are in arbitrary units and error bars are s.e.m.

Then, we found a significant main effect of the gift type (romantic vs. non-romantic) on left ACC activity at the voxel-level but not at the cluster-level (Fig 4A, Table 1). A pairwise comparison between the gift types from the liked giver showed significant difference in activation in this area both at the voxel- and cluster-level (Fig 4B, Table 1). In contrast, we found no significant difference for a pairwise comparison between the gift types from the uninteresting giver. Taken together, the preference for a giver strongly modulates ACC activity while judging romantic compared to non-romantic gifts. To confirm this, we calculated parameter estimates of the ROI in this area across the four target conditions (peak voxel derived from the main effect in MNI: –8/40/22, Fig 4C).

We found no significant interaction of the giver and gift type.

#### Psychophysiological interaction (PPI) analysis

**Fig 5:**
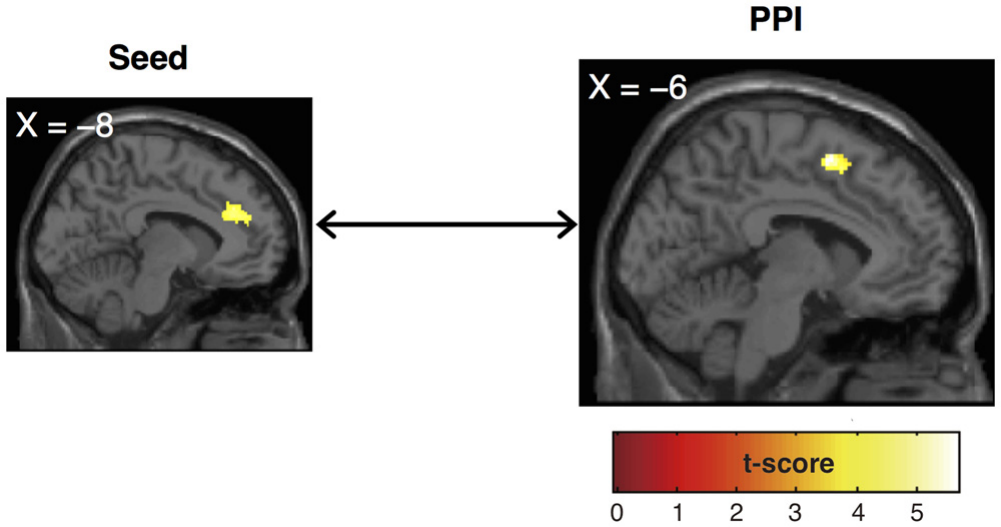
Functional connectivity from the left ACC seed. Significant functional connectivity from the left ACC (seed) was found in the left SMA/dACC in PPI analysis. Activation of PPI is displayed at a threshold of p < 0.001, uncorrected at the voxel-level.

We explored functional connectivity of the left ACC, resultant of a pairwise comparison between the gift types from the liked giver (peak voxel in MNI: –8/36/30). The target contrast for PPI was a pairwise comparison between the givers for romantic gifts (i.e., the romantic gifts from the liked vs. the uninteresting giver)—we investigated functional connectivity in the specific condition in which a female judges a romantic gift from the liked male giver. We found connectivity with the left supplementary motor area/dorsal ACC (SMA/dACC) (peak voxel in MNI: –6/6/56, Z-score = 4.36, cluster size = 89 contiguous voxels, Fig 5).

## Discussion

To our knowledge, this is the first fMRI study to clarify the neural correlates of how preferences for the giver modulate understanding of the gift’s meaning. We found two-fold neural processing for value judgments of gifts. First, preferences for the giver modulated activation in the caudate. Second, while processing of the social meaning of the gifts, preferences for the giver selectively modulated activity in ACC, a cortical area in the reward system. We suggest that the ACC is a preference-dependent detector of the social meaning of material goods in gift-judgment situations.

Our behavioral results showed that preferences for the giver and gift type both affected the gifts’ value from the liked giver. Contrarily, only the indifference toward the giver, and not the gift type, affected the value of a gift from the uninteresting giver. These findings suggest the two-fold mechanisms in value judgments for gifts: gift recipients first consider who the giver is; they then consider what the gift means only if they like the giver. The fact that the gift type differentiated the gift’s value only from the liked giver suggests a modulatory role of preferences for the giver from the opposite sex in understanding of the gifts’ social meaning.

We found the same pattern in fMRI results. First, the attitude toward the liked giver activated the caudate. The gift type (romantic or non-romantic) did not affect activity in this area. Preference for a desired person activates the dopamine-rich reward circuits such as the ventral tegmental area, nucleus accumbens, and caudate [2,9–11]. Importantly, the caudate activation observed in our study was not induced by passively viewing the liked but by receiving gifts from the liked giver. We showed this by subtracting activity for the control tasks (passively viewing the giver and pressing buttons) from that for the target tasks. Preferences for the liked giver and detection of rewards (gifts) therefore modulate activity in the caudate.

Regarding the social meaning of the gifts, romantic gifts *per se* can be a reward, because we found a weak activation in the ACC for the romantic, relative to the non-romantic gifts. This ACC activity, albeit weak, is independent of the giver and suggests that this area attends to the interpretation of the social meaning of the gifts. Previous studies showed that ACC activity reflects increased autonomic nervous activity and attention levels [30]. Importantly, preferences for the liked giver profoundly modulated ACC activity in response to romantic gifts. Contrarily, we did not find this modulation for the uninteresting giver. This giver-dependent activation pattern suggests that understanding of the social meaning is strongly influenced by the givers and that the female participants do not dismiss the social meaning solely by speculating on the feelings of the givers. Our interpretation is based on the fact that we instructed the participants only to focus on judging the attractiveness of the gifts without considering the givers. If the participants had simply speculated on the feelings of the givers, we would also have found specific brain activation in response to romantic gifts from uninteresting givers. Additionally, the ACC is also involved in preferences for a partner [9,10]. Preferences for the liked giver therefore increase autonomic arousal and attention levels, and the area selectively detects socially meaningful rewards (romantic gifts).

A more logical understanding of the ACC function in interpreting the social meaning of the gifts can also be achieved from another perspective. Gifts on specific social occasions contain rewarding properties. These properties are computed in the brain to assess motivational relevance in decision-making [8]. Highly rewarding gifts (i.e., romantic gifts) therefore provide stronger motivation to the recipients in making value judgments. This reward value computation (i.e., to determine whether the object of choice is valuable) is performed in the ACC [31]. Additionally, the ACC uses reward value to choose optimal behavior [32–35]. Lesions in this area impair this reward-guided behavior in both monkeys and humans [36, 37]. The ACC thus plays a vital role in interpreting the appropriate reward value (i.e., social meaning of the gifts) in decision-making.

Further, our finding of functional connectivity between the ACC and SMA/dACC suggests that this connectivity integrates monitoring others and emotional and social cognitive factors in decision-making. Monitoring others’ action is important for adaptive behavior in social interactions [38]. A conjunction analysis revealed functional overlap of action monitoring, emotional processing, and social cognitive processing in the dorsal mediofrontal cortex (dMFC) including the ACC, SMA, and dACC [38]. Considering these findings, we found functional connectivity within the dMFC that integrates the giver’s gift-giving behavior (i.e., action monitoring) with preferences for the liked giver and evaluation of the social meaning of the gift (i.e., emotional and social cognitive processing). This integration leads to a judgment of the attractiveness of the gift in positive interpersonal relationships. We performed functional connectivity analysis with the caudate as a seed region but found no significant connectivity. This may be due to the experimental paradigm in which we had only two givers for each participant and did not ask the participants to evaluate their liking for the givers. In spite of this technical limitation, we assume that the caudate, which responds to preferences for the liked giver, would affect social cognitive processing in the ACC indirectly via other brain regions.

There are two other limitations in this study. First, the current setting may be a stereotyped presentation in a given culture. However, the intention behind gifting behavior (e.g., to express love, to reinforce social solidarity, or to conform to a sense of obligation) is universal [6,21,22]. Second, the categorical difference between the romantic and non-romantic gifts may not always be distinct, because we did not ask each fMRI participant to rate the social meaning of gifts. The reasons for this are mentioned previously (see Materials and Methods). Additionally, we made the task as realistic as possible—we did not use goods such as abstract shapes or blank boxes as a non-romantic gift because people hardly experience to receive such objects.

This study may have an implication for understanding of how humans evolved sophisticated social interactions by using goods. The neural gender difference will also be a scope of interest. Our study may also have clinical implications. People with schizophrenia have difficulty with a task that requires recognition of emotions of the self and others [39]. They are impaired in social interactions [40], and they do not appreciate the rewarding character of courtship relationships [41]. Because the gift-judgment task in this study requires the faculty of recognizing emotions of the self and others in courtship relationships, it may provide another opportunity to investigate social dysfunction in schizophrenia.

## Conclusions

This is the first fMRI study of the neural processing underlying how preferences for the giver modulate understanding of a gift’s meaning. The female gift recipients first consider who the giver is and then consider what the gift means only if they like the giver. The attitude toward the liked but not the uninteresting giver modulates ACC activity to recognize the gift’s social meaning. This preference-dependent detection of the social meaning may enable the recipients to assess the motivational relevance of gifts to make decisions in courtship relationships. Additional studies are needed to further determine the ACC function in other courtship situations.

## Supporting Information

S1 Table Attractiveness of the material goods *per se* and their romantic value as a gift from a male giver (romantic gifts). (DOC)

S2 Table Attractiveness of the material goods *per se* and their romantic value as a gift from a male giver (non-romantic gifts). (DOC)
