# Supplementary material for: Women’s Preference for a Male Acquaintance Enhances Social Reward Processing of Material Goods in the Anterior Cingulate Cortex
Source: PLoS One. 2015 Aug 24;10(8):e0136168. doi: 10.1371/journal.pone.0136168 (PMC4547715; doi:10.1371/journal.pone.0136168)
Supplement: S1 Table — (DOC) [file pone.0136168.s001.doc]

**S1 Table. Attractiveness of the material goods *per se* and their romantic value as a gift from a male giver (romantic gifts).**

| No. | Material goods | Attractiveness (1–9) | | | Romantic value (1–3) | | |
| --- | --- | --- | --- | --- | --- | --- | --- |
|  |  | mean | ± | s.d. | mean | ± | s.d. |
| 1 | Aroma candle | 5.35 | ± | 2.59 | 2.39 | ± | 0.88 |
| 2 | At-home beauty tool | 5.42 | ± | 2.42 | 2.19 | ± | 0.79 |
| 3 | Bath soaps | 6.26 | ± | 2.41 | 2.68 | ± | 0.48 |
| 4 | Books | 6.16 | ± | 2.35 | 2.52 | ± | 0.72 |
| 5 | Bracelet | 6.39 | ± | 2.22 | 2.81 | ± | 0.60 |
| 6 | Brooches | 3.74 | ± | 2.31 | 2.52 | ± | 0.77 |
| 7 | Cashmere gloves | 4.06 | ± | 2.16 | 2.61 | ± | 0.72 |
| 8 | City bike | 5.32 | ± | 2.10 | 2.29 | ± | 0.86 |
| 9 | Earrings | 4.74 | ± | 3.08 | 2.77 | ± | 0.62 |
| 10 | Fancy hat | 4.13 | ± | 2.06 | 2.35 | ± | 0.84 |
| 11 | Fancy music box | 5.32 | ± | 2.73 | 2.65 | ± | 0.61 |
| 12 | Fancy pen case | 6.32 | ± | 2.27 | 2.13 | ± | 0.92 |
| 13 | Fine-knit sweater | 6.00 | ± | 2.14 | 2.42 | ± | 0.72 |
| 14 | Flower bouquet | 6.06 | ± | 2.11 | 2.84 | ± | 0.52 |
| 15 | Fountain pen | 5.03 | ± | 2.39 | 2.35 | ± | 0.88 |
| 16 | Greeting cards | 4.61 | ± | 2.32 | 2.32 | ± | 0.83 |
| 17 | Hug pillow | 6.48 | ± | 2.22 | 2.55 | ± | 0.68 |
| 18 | Key wallet | 4.23 | ± | 2.32 | 2.08 | ± | 0.89 |
| 19 | Kitchen apron | 3.26 | ± | 2.03 | 2.12 | ± | 0.86 |
| 20 | Knit scarf | 5.65 | ± | 2.48 | 2.68 | ± | 0.70 |
| 21 | Lipstick | 5.81 | ± | 2.30 | 2.68 | ± | 0.60 |
| 22 | Makeup pouch | 5.45 | ± | 2.22 | 2.39 | ± | 0.84 |
| 23 | Good-luck bracelet | 4.65 | ± | 2.71 | 2.19 | ± | 0.91 |
| 24 | Nail polish | 5.71 | ± | 2.64 | 2.23 | ± | 0.84 |
| 25 | Perfume | 4.84 | ± | 3.06 | 2.61 | ± | 0.76 |
| 26 | Picture frames | 4.35 | ± | 2.23 | 2.13 | ± | 0.85 |
| 27 | Premium headphones | 4.81 | ± | 2.41 | 2.10 | ± | 0.79 |
| 28 | Rough diamonds | 6.00 | ± | 2.90 | 2.71 | ± | 0.64 |
| 29 | Stuffed toy bear | 6.39 | ± | 2.12 | 2.77 | ± | 0.62 |
| 30 | Tea cups | 4.71 | ± | 2.15 | 2.26 | ± | 0.89 |
