# Supplementary material for: Women’s Preference for a Male Acquaintance Enhances Social Reward Processing of Material Goods in the Anterior Cingulate Cortex
Source: PLoS One. 2015 Aug 24;10(8):e0136168. doi: 10.1371/journal.pone.0136168 (PMC4547715; doi:10.1371/journal.pone.0136168)
Supplement: S2 Table — (DOC) [file pone.0136168.s002.doc]

**S2 Table. Attractiveness of the material goods *per se* and their romantic value as a gift from a male giver (non-romantic gifts).**

| No. | Material goods | Attractiveness (1–9) | | | Romantic value (1–3) | | |
| --- | --- | --- | --- | --- | --- | --- | --- |
|  |  | mean | ± | s.d. | mean | ± | s.d. |
| 1 | Biscuits | 5.10 | ± | 2.33 | 1.68 | ± | 0.79 |
| 2 | Clear file folder | 4.32 | ± | 2.37 | 1.03 | ± | 0.18 |
| 3 | Coins | 4.65 | ± | 2.35 | 1.58 | ± | 0.76 |
| 4 | Detergent | 4.23 | ± | 1.76 | 1.45 | ± | 0.77 |
| 5 | Electric dictionary | 6.03 | ± | 2.18 | 2.03 | ± | 0.80 |
| 6 | Electric fan | 4.74 | ± | 2.59 | 1.42 | ± | 0.62 |
| 7 | Electric kettle | 5.03 | ± | 2.04 | 1.87 | ± | 0.81 |
| 8 | Hair brush | 4.58 | ± | 1.96 | 1.29 | ± | 0.59 |
| 9 | Hair dryer | 5.29 | ± | 2.07 | 1.45 | ± | 0.62 |
| 10 | Hand cream | 5.52 | ± | 2.34 | 1.84 | ± | 0.86 |
| 11 | Houseplant | 4.84 | ± | 2.77 | 2.03 | ± | 0.84 |
| 12 | Jam | 5.35 | ± | 2.30 | 1.87 | ± | 0.92 |
| 13 | Japanese fan | 4.45 | ± | 2.42 | 1.71 | ± | 0.90 |
| 14 | Juice | 6.58 | ± | 2.00 | 1.74 | ± | 0.73 |
| 15 | Letter writing set | 4.68 | ± | 2.45 | 1.58 | ± | 0.81 |
| 16 | Lunch box | 5.71 | ± | 2.15 | 1.71 | ± | 0.82 |
| 17 | Magazine | 5.26 | ± | 2.14 | 1.39 | ± | 0.50 |
| 18 | Memo pad | 4.19 | ± | 2.09 | 1.26 | ± | 0.51 |
| 19 | Notebook | 4.68 | ± | 1.97 | 1.39 | ± | 0.56 |
| 20 | Office chair | 4.35 | ± | 1.94 | 1.71 | ± | 0.82 |
| 21 | Painting | 4.19 | ± | 2.51 | 1.97 | ± | 0.80 |
| 22 | Pencils | 5.23 | ± | 2.17 | 1.48 | ± | 0.72 |
| 23 | Photo album | 4.45 | ± | 2.57 | 1.45 | ± | 0.72 |
| 24 | Moneybox | 4.35 | ± | 2.64 | 1.23 | ± | 0.56 |
| 25 | Shampoo | 5.74 | ± | 2.05 | 1.45 | ± | 0.68 |
| 26 | Sunscreen | 6.58 | ± | 2.22 | 1.68 | ± | 0.79 |
| 27 | T-shirt | 4.68 | ± | 2.26 | 1.68 | ± | 0.83 |
| 28 | Tissue paper | 4.29 | ± | 2.15 | 1.26 | ± | 0.44 |
| 29 | Towel | 4.74 | ± | 2.27 | 1.68 | ± | 0.87 |
| 30 | Wall clock | 4.32 | ± | 2.41 | 1.65 | ± | 0.75 |
